# Evaluation of the Use of Home Blood Pressure Measurement Using Mobile Phone-Assisted Technology: The iVitality Proof-of-Principle Study

**DOI:** 10.2196/mhealth.5485

**Published:** 2016-06-13

**Authors:** Liselotte W Wijsman, Edo Richard, Ricardo Cachucho, Anton JM de Craen, Susan Jongstra, Simon P Mooijaart

**Affiliations:** ^1^ Leiden University Medical Center Department of Gerontology & Geriatrics Leiden Netherlands; ^2^ Radboud University Medical Center Department of Neurology Nijmegen Netherlands; ^3^ Academic Medical Center Amsterdam Department of Neurology Amsterdam Netherlands; ^4^ Leiden Institute of Advanced Computer Sciences University of Leiden Leiden Netherlands; ^5^ Institute of Evidence-Based Medicine in Old Age Leiden Netherlands

**Keywords:** mobile phone, home-based measurements, hypertension, dementia

## Abstract

**Background:**

Mobile phone-assisted technologies provide the opportunity to optimize the feasibility of long-term blood pressure (BP) monitoring at home, with the potential of large-scale data collection.

**Objective:**

In this proof-of-principle study, we evaluated the feasibility of home BP monitoring using mobile phone-assisted technology, by investigating (1) the association between study center and home BP measurements; (2) adherence to reminders on the mobile phone to perform home BP measurements; and (3) referrals, treatment consequences and BP reduction after a raised home BP was diagnosed.

**Methods:**

We used iVitality, a research platform that comprises a Website, a mobile phone-based app, and health sensors, to measure BP and several other health characteristics during a 6-month period. BP was measured twice at baseline at the study center. Home BP was measured on 4 days during the first week, and thereafter, at semimonthly or monthly intervals, for which participants received reminders on their mobile phone. In the monthly protocol, measurements were performed during 2 consecutive days. In the semimonthly protocol, BP was measured at 1 day.

**Results:**

We included 151 participants (mean age [standard deviation] 57.3 [5.3] years). BP measured at the study center was systematically higher when compared with home BP measurements (mean difference systolic BP [standard error] 8.72 [1.08] and diastolic BP 5.81 [0.68] mm Hg, respectively). Correlation of study center and home measurements of BP was high (*R*=0.72 for systolic BP and 0.72 for diastolic BP, both *P*<.001). Adherence was better in participants measuring semimonthly (71.4%) compared with participants performing monthly measurements (64.3%, *P*=.008). During the study, 41 (27.2%) participants were referred to their general practitioner because of a high BP. Referred participants had a decrease in their BP during follow-up (mean difference final and initial [standard error] −5.29 [1.92] for systolic BP and −2.93 [1.08] for diastolic BP, both *P*<.05).

**Conclusion:**

Mobile phone-assisted technology is a reliable and promising method with good adherence to measure BP at home during a 6-month period. This provides a possibility for implementation in large-scale studies and can potentially contribute to BP reduction.

## Introduction

High blood pressure contributes to the global burden of disease, accounting for 9.4 million deaths per year [1]. With a prevalence as high as 78% among the 65-plus population in Europe, it is one of the most common chronic conditions in primary care [2]. Although the prevalence is predicted to further increase over the coming years, only 70% of all hypertensive patients are aware of having hypertension [3,4]. In spite of widely available effective ways to reduce blood pressure, rates of hypertension control are still far from optimal [2,5-7]. In one study including 5296 participants, blood pressure control was achieved in only 30% of patients, who were aged 60 years or older [8].

The increasing availability of the Internet, mobile phones, and health sensors provides the potential to interactively administer health interventions at home. Recent surveys show that at least 75% of the European population uses the Internet on a regular basis, with almost half of them using a mobile phone to access the Internet (smartphone) [9]. Adults aged 65 years and older are the fastest-growing group of Internet users [10]. Older adults have a high interest in self-assessment health tools [11]. This enables people to measure blood pressure at home, with the potential of direct feedback and treatment adjustments. Furthermore, previous studies show that home blood pressure measurements, when compared with clinic blood pressure measurements, are in fact a stronger prognostic indicator of cardiovascular events [12-14]. It could therefore be effective to identify patients at risk of cardiovascular events and thereby prevent the occurrence of cardiovascular complications [12,15]. In addition, it provides a potential for large-scale implementation and data collection. However, data on feasibility of long-term home blood pressure measurements, using the Internet and mobile phones, are scarce [16-18].

The aim of this proof-of-principle study was to evaluate home blood pressure measurements using mobile phone-assisted technology. For this, we investigated (1) the association between study center and home blood pressure; (2) the adherence to perform home blood pressure measurements according to a monthly or semimonthly measurement protocol; and (3) referrals, treatment consequences and blood pressure reduction after a raised home blood pressure was diagnosed.

## Methods

### Study Design

iVitality is a Web-based research platform that consists of a Website, a mobile phone-based app, and sensors that are connected with or already integrated in the mobile phone to measure blood pressure [19]. This iVitality study is a proof-of-principle study in which participants were randomized to perform home blood pressure measurements according to a monthly or semimonthly measurement protocol, during a period of 6 months. The different measurement protocols are described in more detail in the “Follow-Up Measurements” paragraph below.

### Study Participants

We chose to perform this study in people with a parental history of dementia because (1) they have a higher risk of both hypertension and dementia, making them a potentially suitable target group for large-scale preventive studies and (2) they are highly motivated to participate in preventive studies [13,14,15]. Other inclusion criteria were (1) age 50 years and older; (2) familiar with and in possession of a mobile phone with iOS or Android (version, 2.3.3 or higher) software; and (3) motivated to measure health characteristics at home several times a month, during a 6-month period. Exclusion criteria were a medical diagnosis of dementia and/or any other cognitive disorder and a medical history of stroke and/or transient ischemic attack.

Participants were recruited through advertisements in memory outpatient clinics, nursing homes, general practices, and on the Website, and in the newsletter of the Dutch Alzheimer Foundation (Alzheimer-Nederland). If all of the inclusion criteria were met, participants received detailed study information in print. They visited the study center at Leiden University Medical Center or Academic Medical Center Amsterdam at baseline, where they received information about the study and baseline measurements were performed by a study physician or research nurse. Written informed consent was obtained from all participants. The medical ethical committee of Leiden University Medical Center, the Netherlands, approved the study.

### Baseline Measurements

Enrolment and follow-up took place from September 2013 to January 2015. In preparation for the first visit to the study center, all participants completed a Web-based questionnaire on education, medical history, and medication use. During the visit to the study center, detailed information about the iVitality app and instructions on how to use it were given. History of hypertension and medication use was self-reported. Blood pressure was measured twice at baseline on the upper left arm, in sitting position with a fully automatic electronic blood pressure monitor. Participants were instructed in the use of the home blood pressure monitor.

### Follow-Up Measurements

During a 6-month period, participants received automatic messages on preprogrammed days at self-chosen time points on their mobile phone, which reminded them to measure blood pressure. Participants with an Android mobile phone used an A&D blood pressure monitor (A&D Company, Ltd; model UA 767 Bluetooth) which was connected to the mobile phone and automatically transferred the results to the iVitality app by Bluetooth [20]. Participants with an iPhone used an OMRON (OMRON Healthcare Company, Ltd, model M6W and M6AC [HEM-7322-E]); they manually typed their blood pressure values and heart rate in the iVitality app [21]. During the first week and the last week of the study, all participants performed blood pressure measurements according to the guideline of the European Society of Hypertension [13]. In short, participants were asked to measure their blood pressure twice at both morning and evening, at least for 4 days during the first week of the study, with day 1 being discarded [13]. For the rest of the study period, blood pressure was measured according to 2 different study protocols, to which participants were randomly assigned by a computerized program at baseline. Randomization was performed in a 1:1 manner stratified for sex. In the monthly protocol, participants performed measurements in the morning and evening of 2 consecutive days. In the semimonthly protocol, blood pressure was measured in the morning and evening of only one day. Blood pressure was measured twice at each measurement; the mean of both measurements was used. Reminders to perform blood pressure measurements were sent the evening before the measurement day and at the actual day on which the participant was expected to perform the blood pressure measurements. When participants did not perform their blood pressure measurements, they received a reminder the day after. This reminder was sent automatically by the Website of the iVitality research platform, and therefore, was a standardized procedure. The measured blood pressure was sent as a message to the mobile phone. Blood pressure measurements were also graphically visible in the app. Participants with a mean systolic home blood pressure above 135 mm Hg and/or 85 mm Hg for diastolic home blood pressure during these days were considered as possibly having hypertension and therefore referred to their general practitioner (GP) [13].

### Statistical Analysis

Characteristics of the study participants are reported as mean with standard deviation for continuous variables and as number with percentage for categorical variables. We used Pearson’s correlation coefficient to calculate the correlation between home and study center blood pressure measurements. To investigate agreement between study center and home blood pressure measurements, we computed the mean and the difference in study center and home blood pressure measurements and visualized this in a Bland–Altman plot.

Adherence was defined as the actual performance of all blood pressure measurements within 1 week of the time point they were expected to perform their measurements and for which the participant received reminders through the mobile phone app. For each participant, we calculated the percentage of adherence during follow-up. Difference in adherence between the monthly and semimonthly measurement protocol was assessed using a Mann–Whitney *U* test. In this proof-of-principle study, only participants who completed the 6-month period were used in the primary analysis. In a sensitivity analysis, we included all participants who were included at baseline.

We investigated the difference in blood pressure during the first week and the final week after 6 months using a paired *t* -test. For both blood pressure during the first week and final blood pressure, we calculated the mean values of all blood pressure measurements performed during the first and last week, with day 1 of both weeks being discarded [13].

All analyses were performed using SPSS (version 22.0.0, SPSS Inc., Chicago, IL, USA).

## Results

### Baseline characteristics

**Figure 1:**
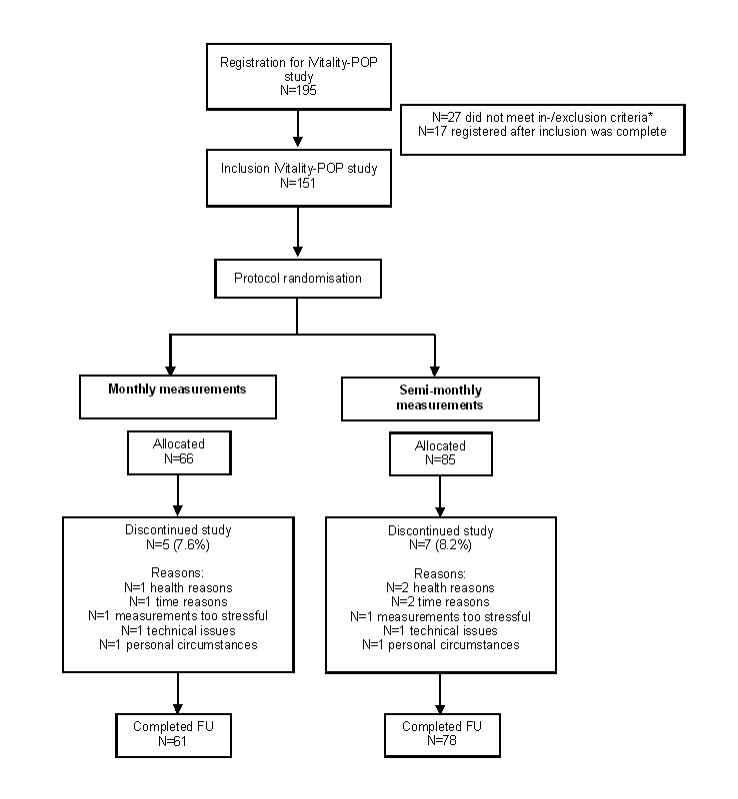
Flowchart of study participants. *Reasons why participants did not meet in-/exclusion criteria were as follows: n=4 did not have the correct software version of the mobile phone; n=6 did not have parents with dementia; n=6 were on holidays during the inclusion period; n=7 were not interested to participate after reading the study information; n=4 because of other reasons.

Figure 1 shows the inclusion flowchart of participants. A total of 195 participants registered on the Web to participate. Of those, 27 did not meet inclusion criteria and 17 registered after recruitment had been completed because of a time lag between registration on the Web and baseline visits. Our study population therefore included 151 participants. A number of 66 (43.7%) participants were assigned to perform blood pressure measurements at a monthly interval; 85 (56.3%) participants were assigned to perform semimonthly blood pressure measurements (Figure 1).

Baseline characteristics are shown in Table 1. Mean age was 57.3 (standard deviation [SD] 5.3) years; 107 (70.9%) participants were female. Of all participants, 56 (37.1%) used iPhone and 59 (39.1%) used Samsung. Mean systolic and diastolic blood pressure measured at the study center was 137.8 (SD 18.2) and 85.4 (SD 10.8) mm Hg, respectively. Participants within the monthly protocol had a higher body mass index, systolic blood pressure, and diastolic blood pressure at baseline (Multimedia Appendix 1). A number of 32 (21.2%) participants had a history of hypertension and used antihypertensive medication, most commonly diuretics (15 participants [46.9% of hypertensive participants]). This did not differ between the monthly and semimonthly protocol.

**Table 1:** Baseline characteristics of iVitality participants.^a,b^

Demographics		All participants (N=151)
Age (years)		57.3 (5.3)
Female, n (%)		107 (70.9)
Body mass index		26.4 (4.0)
Highest education level, n (%)^c^		
	Low	16 (10.6)
	Middle	44 (29.1)
	High	88 (58.3)
Study center, n (%)		
	Academic Medical Center Amsterdam	55 (36.4)
	Leiden University Medical Center	96 (63.6)
Type of phone, n (%)		
	iPhone	56 (37.1)
	Samsung	59 (39.1)
	HTC	15 (9.9)
	Other	21 (13.9)
Blood pressure		
	Systolic blood pressure (mm Hg)	137.8 (18.2)
	Diastolic blood pressure (mm Hg)	85.4 (10.8)
	Heart rate (bpm)	67.2 (10.2)
Vascular risk factors, n (%)		
	History of hypertension	32 (21.2)
	History of diabetes mellitus	2 (1.3)
	History of MI	4 (4.6)
	History of arrhythmia	11 (7.3)
	History of heart failure	3 (2.0)
	Hypercholesterolemia	14 (9.3)
	Current smoker	14 (9.3)
Antihypertensive medication, n (%)		
	Diuretics	15 (9.9)
	ACE inhibitors	6 (4.0)
	Beta-blockers	11 (7.3)
	Calcium antagonists	6 (4.0)
	Other	9 (6.0)
No of antihypertensive medication, n (%)		
	1	21 (65.6)
	2 or more	11 (34.4)

^a^Data represent mean (standard deviation) unless stated otherwise

^b^Abbreviations: MI, myocardial infarction; MMSE, mini-mental state examination.

^c^Missing data for n=3 participants. Low: primary education, lower education, MAVO/MULO. Intermediate: high general secondary education (HAVO, HBS), Preparatory Scientific Education (VWO), intermediate professional education (MBO). High: higher professional education (HBO), academic education (university).

### Main findings

**Figure 2:**
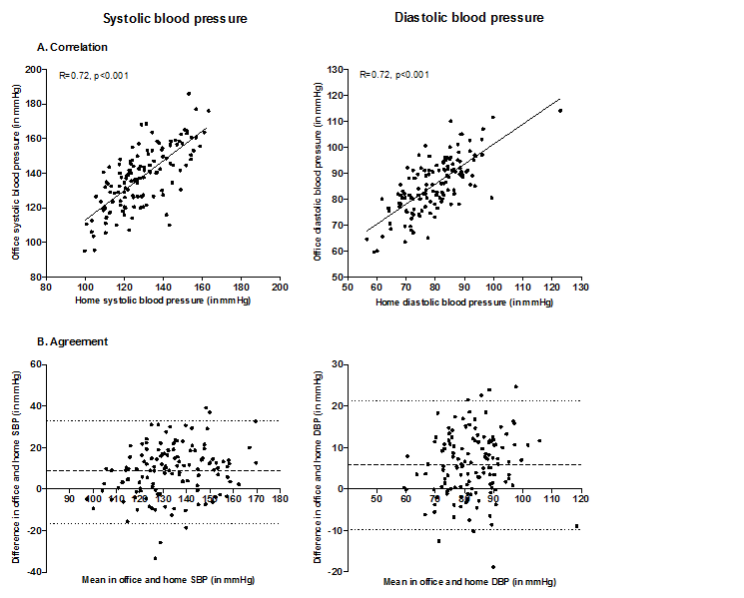
Association between blood pressure measurements at home and in the study center. Abbreviations: R, Pearson’s correlation coefficient; SBP, systolic blood pressure; DBP, diastolic blood pressure. Panel A shows the home blood pressure (mean of two consecutive measurements in both morning and evening at day 2, 3 and 4, x-axis) and corresponding study center blood pressure measurements (mean value of two consecutive measurements, y-axis) for each participant. Panel B shows the agreement between study center and home systolic and diastolic blood pressure measurements.

The association between study center and home blood pressure during the first week is shown in Figure 2. The correlation between study center and home measurements was high for both systolic (*R*=0.72, *P*<.001) and diastolic blood pressure (*R*=0.72, *P*<.001; panel A). Systolic blood pressure at the study center was systematically 8.72 mm Hg higher (standard error [SE] 1.08) and diastolic blood pressure was 5.81 (0.68) mm Hg higher when compared with home blood pressure measurements (panel B). The Bland–Altman plot shows that the difference between the measurements was randomly distributed over the mean of the measurements, indicating that there was no systematic bias in agreement between the study center and home measurements. The 95% limits of agreement for the comparison were −16.82 to 32.82 mm Hg for systolic blood pressure and −9.71 to 21.33 mm Hg for diastolic blood pressure.

**Figure 3:**
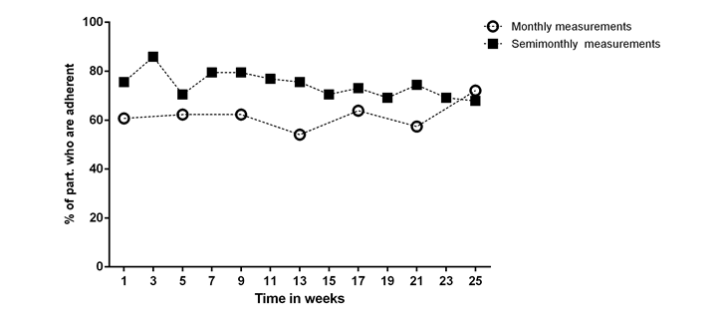
Adherence of participants to monthly and semi-monthly measurement protocol. Data represent the percentage of participants who are adherent to perform the expected blood pressure measurements.

Adherence to the monthly and semimonthly blood pressure measurement protocols is shown in Figure 3. In total, 12 participants did not complete follow-up: 5 (7.6%) participants in the monthly measurement protocol and 7 (8.2%) participants in the semimonthly measurement protocol. Median adherence to perform blood pressure measurements was 71.4% (Figure 3). Participants performing semimonthly blood pressure measurements were more adherent (median adherence 71.4%) when compared with participants performing monthly blood pressure measurements (64.3%, *P*=.008). There was no difference in adherence between participants who entered their blood pressure measurements manually and participants who used a Bluetooth connection to transfer the measurements (data not shown). Furthermore, a sensitivity analysis in which we investigated the adherence of all 151 participants who were included at baseline, showed similar results: adherence was higher in participants who performed semimonthly blood pressure measurements (median adherence 85.7%) when compared with participants performing monthly measurements (71.4%, *P*=.002; Multimedia Appendix 2). Discontinuation was highest within the first weeks of follow-up for both measurement protocols.

Table 2 presents the difference in final and initial home blood pressure measurements. Among all participants, there was no difference between final and initial blood pressure, for both systolic (mean difference [SE] −1.30 [1.10] mm Hg, *P*=.240) and diastolic (−0.90 [0.51] mm Hg, *P*=.081) blood pressure. There were 41 out of 151 (27.2%) participants who were referred to their GP because of a high blood pressure, of whom 35 (85.2%) actually visited their GP. In referred participants, blood pressure decreased significantly during the study, both systolic (mean difference [SE] −5.29 [1.92] mm Hg, *P*=.011) and diastolic (−2.93 [1.08] mm Hg, *P*=.012). Furthermore, no difference was found between final and initial blood pressure for both systolic and diastolic blood pressure between the monthly and semimonthly protocol (data not shown). In 7 out of 41 (17.1%) participants, blood pressure lowering medication had been started or changed.

**Table 2:** Difference between the first and last home blood pressure measurement.^a,b^

		Systolic blood pressure				Diastolic blood pressure			
		First	Last	Diff. (SE)	*P*-value	First	Last	Diff. (SE)	*P*-value
All participants^c^		128.22 (1.45)	126.92 (1.31)	−1.30 (1.10)	.240	79.30 (0.90)	78.40 (0.73)	−0.90 (0.51)	.081
By protocol									
	Monthly measurements, n=45	128.23 (2.25)	128.15 (2.25)	−0.08 (1.98)	.968	80.08 (1.38)	78.87 (1.18)	−1.21 (0.84)	.156
	Biweekly measurements, n=58	128.20 (1.91)	125.96 (1.54)	−2.25 (1.20)	.068	78.70 (1.20)	78.02 (0.92)	−0.67 (0.64)	.304
By referral									
	Not referred, n=78	122.61 (1.27)	122.59 (1.28)	−0.02 (1.29)	.987	76.71 (0.91)	76.45 (0.80)	−0.25 (0.56)	.654
	Referred, n=25	145.72 (1.96)	140.43 (1.91)	−5.29 (1.92)	.011	87.22 (1.66)	84.44 (1.02)	−2.93 (1.08)	.012
	Referred and visited GP, n=23	146.02 (2.03)	140.98 (2.00)	−5.04 (2.07)	.023	87.22 (1.66)	84.44 (1.02)	−2.79 (1.17)	.027
	Referred and changed/started BP med., n=5	153.73 (3.93)	141.62 (4.83)	−12.12 (6.65)	.142	90.62 (2.64)	83.79 (1.55)	−6.83 (2.29)	.080

^a^Data represent the mean difference (standard error) in mm Hg of final and initial blood pressure.

^b^Diff, difference; SE, standard error; GP, general practitioner; BP, blood pressure.

^c^Missing data for n=48 participants.

## Discussion

### Principal Findings

This proof-of-principle study, in which we evaluated the feasibility of home blood pressure measurements during a 6-month intervention period using mobile phone-assisted technology, has 3 main findings. First, study center and home blood pressure were highly correlated, although blood pressure measured at the study center was systematically higher when compared with home blood pressure. Second, adherence of all participants to perform blood pressure measurements was high and persisted during 6 months with better adherence in participants measuring semimonthly compared with participants who performed monthly measurements. Third, in participants who were referred to their GP because of a high blood pressure, systolic and diastolic blood pressure decreased significantly during the study, especially for those who started medication.

Our finding that systolic and diastolic blood pressure at the study center was systematically higher when compared with home blood pressure measurements is in line with previous literature [22]. A well-known explanation for this is the “white-coat effect,” meaning that blood pressure is higher because of stress and anxiety that patients experience during a clinical setting [22,23]. Literature shows that home blood pressure measurements, instead of office or clinic blood pressure measurements, are in fact a stronger prognostic indicator of cardiovascular events and even have their own (lower) reference values [12-14]. A participant-level meta-analysis including 5008 participants (mean age 57 years, not treated with antihypertensive medication), showed that in participants with an optimal office blood pressure (<120/<80 mm Hg), a 10-mm Hg higher systolic home blood pressure increased the risk of any cardiovascular event by nearly 30% [12]. In addition, previous studies on cost-effectiveness show that compared with usual care, home blood pressure monitoring is very useful for reducing health care costs. In view of the low burden of measuring and established treatment options, home blood pressure monitoring could therefore be an important strategy to further prevent cardiovascular complications, especially in people at risk [13].

Previous studies on adherence to perform home blood pressure measurements show similar results to our findings [17,24]. In a study on telemonitoring including 213 hypertensive patients, who were asked to measure their blood pressure at least 6 times a week during 6 months, mean adherence was 73% [17]. Another study including only patients with heart failure (mean age 61 years) showed adherence of 55% [24]. Furthermore, in this study, we found that participants using the semimonthly measurement protocol showed higher adherence compared with participants using the monthly measurement protocol. A possible explanation could be that the fact that participants received a reminder twice a month (instead of once a month), might have kept participants more engaged in the study and therefore increased their adherence [19]. Furthermore, the burden of measuring for 1 day every 2 weeks may have been perceived lower than measuring during 2 consecutive days, albeit with monthly intervals.

Home-based blood pressure measurements using mobile phone-based technology may have several potential opportunities. First, other parameters derived from repeated blood pressure measurements, can easily be calculated, especially in a home-based setting. An example of such a parameter is blood pressure variability, of which we recently showed its association with cognitive decline [25]. Second, the combination with other parameters and measurements may reveal additional targets for blood pressure control. Physical activity, sleep, and other lifestyle factors can be measured using a mobile phone. This offers the potential for interventions, for instance aimed at increasing physical activity, that also beneficially affect blood pressure. The iVitality platform offers the opportunity to assess these lifestyle factors. Third, mobile phone-based technology might be a cost-effective alternative in the control of hypertension. It was previously shown that ambulatory blood pressure monitoring as a diagnostic strategy for hypertension saves costs, mainly because additional costs from ambulatory monitoring are counterbalanced by cost savings from better targeted treatment [26]. As mobile phone-based technology only requires a standard blood pressure monitor, which is much cheaper when compared with an ambulatory blood pressure monitor, we believe it has the potential of saving health care costs.

In our study, blood pressure was lower at the end of follow-up when the participant was referred to the GP because of a high blood pressure at baseline. There are 2 possible explanations for this finding. First, the decrease in blood pressure may be the result of regression to the mean. This phenomenon occurs when repeated measurements tend to be followed by measurements that are closer to the mean. Although our baseline measurements were defined on repeated blood pressure measurements, it is still expected that the mean blood pressure during follow-up will go down, owing to regression toward the mean. Second, it may reflect a true effect of monitoring and subsequent treatment of blood pressure. Blood pressure lowering interventions by the GP and higher awareness of participants may all have contributed to a lower blood pressure. Although the fact that the effect on blood pressure was highest in those who initiated medication suggests the second explanation, we have not collected enough information on interventions in this proof-of-principle study to draw definite conclusions. An adequately powered randomized controlled trial may help to establish the effects of the interventions.

For this proof-of-principle study, we selected highly motivated participants with a parental history of dementia. This may have introduced a selection bias toward better adherence and treatment effects, which reduces the external validity for other, broader defined populations. The strength of this study is that mobile phone technology was used to collect study data on blood pressure. This innovative method reduces the need for face-to-face contact and stimulates self-management. Now that this proof-of-principle study is promising, broader and larger populations can be included in future studies.

### Conclusions

This proof-of-principle study demonstrates that mobile phone-assisted technology can be used as a reliable and promising method to measure blood pressure at home during a 6-month period. This provides a possibility for implementation in large-scale studies and can potentially lead to blood pressure reduction and eventually reduction of cardiovascular disease.

## Appendix

Multimedia Appendix 1 Baseline characteristics of iVitality participants, by protocol.

Multimedia Appendix 2 Adherence of participants to monthly and semimonthly measurement protocol (all participants included).
