# Supplementary material for: Evaluation of the Use of Home Blood Pressure Measurement Using Mobile Phone-Assisted Technology: The iVitality Proof-of-Principle Study
Source: JMIR Mhealth Uhealth. 2016 Jun 13;4(2):e67. doi: 10.2196/mhealth.5485 (PMC4923587; doi:10.2196/mhealth.5485)
Supplement: Multimedia Appendix 1 [file mhealth_v4i2e67_app1.pdf]

	All participants N=151	Monthly measurements N=66	Semi-monthly measurements N=85
**Demographics**			
Age (years)	57.3 (5.3)	56.8 (5.3)	57.6 (5.3)
Female, n (%)	107 (70.9%)	48 (72.7%)	59 (69.4%)
Body mass index	26.4 (4.0)	27.2 (4.3)	25.7 (3.6)
Highest education level, n (%)*			
Low	16 (10.6%)	8 (12.1%)	8 (9.4%)
Middle	44 (29.1%)	18 (27.3%)	26 (30.6%)
High	88 (58.3%)	39 (59.1%)	49 (57.6%)
Study center, n (%)			
Academic Medical Center Amsterdam	55 (36.4%)	22 (33.3%)	33 (38.8%)
Leiden University Medical Center	96 (63.6%)	44 (66.7%)	52 (61.2%)
**Type of phone, n (%)**			
iPhone	56 (37.1%)	21 (31.8%)	35 (41.2%)
Samsung	59 (39.1%)	30 (45.5%)	29 (34.1%)
HTC	15 (9.9%)	6 (9.1%)	9 (10.6%)
Other	21 (13.9%)	9 (13.6%)	12 (14.1%)
**Blood pressure**			
Systolic blood pressure (mmHg)	137.8 (18.2)	141.6 (18.3)	134.9 (17.6)
Diastolic blood pressure (mmHg)	85.4 (10.8)	87.3 (10.2)	83.9 (11.0)
Heart rate (beats per minute)	67.2 (10.2)	67.4 (10.4)	67.0 (10.2)
**Vascular risk factors, n (%)**			
History of hypertension	32 (21.2%)	17 (25.8%)	15 (17.6%)
History of diabetes mellitus	2 (1.3%)	2 (3.0%)	0 (0.0%)
History of MI	4 (4.6%)	2 (3.0%)	2 (2.4%)
History of arrhythmia	11 (7.3%)	3 (4.5%)	8 (9.4%)
History of heart failure	3 (2.0%)	1 (1.5%)	2 (2.4%)
Hypercholesterolemia	14 (9.3%)	4 (6.1%)	10 (11.8%)
Current smoker	14 (9.3%)	5 (7.6%)	9 (10.6%)
**Antihypertensive medication, n (%)**			
Diuretics	15 (9.9%)	9 (13.6%)	6 (7.1%)
Ace-inhibitors	6 (4.0%)	3 (4.5%)	3 (3.5%)
Beta-blockers	11 (7.3%)	6 (9.1%)	5 (5.9%)
Calcium antagonists	6 (4.0%)	2 (3.0%)	4 (4.7%)
Other antihypertensives	9 (6.0%)	3 (4.5%)	6 (7.1%)
**No. antihypertensive medication, n (%)**			
One	21 (65.6%)	10 (15.2%)	11 (12.9%)
Two or more	11 (34.4%)	5 (7.8%)	6 (7.1%)

Data represent mean (standard deviation) unless stated otherwise. Abbreviations: n, number; MI, myocardial infarction; MMSE, mini-mental state examination. *Missing data for n=3 participants. Low: primary education, lower education, MAVO/MULO. Intermediate: high general secondary education (HAVO, HBS), Preparatory Scientific Education (VWO), intermediate professional education (MBO). High: higher professional education (HBO), academic education (university).
