# Supplementary material for: Evaluation of the Use of Home Blood Pressure Measurement Using Mobile Phone-Assisted Technology: The iVitality Proof-of-Principle Study
Source: JMIR Mhealth Uhealth. 2016 Jun 13;4(2):e67. doi: 10.2196/mhealth.5485 (PMC4923587; doi:10.2196/mhealth.5485)
Supplement: Multimedia Appendix 2 [file mhealth_v4i2e67_app2.pdf]

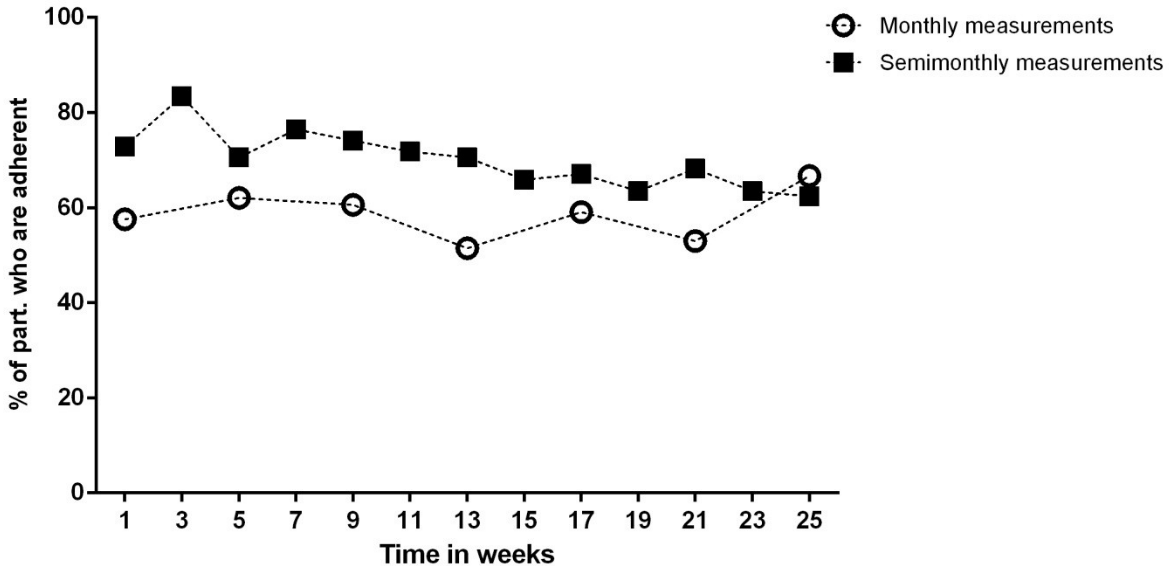

Data represent the percentage of participants who are adherent to perform the expected measurements.
